# Pre-hypertension in Bangladesh: evidence from BDHS 2022

**DOI:** 10.3389/fpubh.2026.1777932

**Published:** 2026-04-10

**Authors:** Kazi Sabbir Ahmad Nahin, Arman Hossen

**Affiliations:** 1 Dr. Bing Zhang Department of Statistics, University of Kentucky, Lexington, KY, United States; 2 Department of Statistics, Miami University, Oxford, OH, United States

**Keywords:** BDHS 2022, pre-hypertension, BMI, random forest, ensemble learning, logistic regression, sociodemographic factors

## Abstract

**Background:**

Hypertension drives pre-mature mortality globally, yet its precursor, pre-hypertension (pre-HTN), remains under-researched in Bangladesh despite offering a critical window for intervention. This study examined the socio-demographic and health-related determinants of pre-hypertension among Bangladeshi adults and evaluated whether machine learning models provide additional predictive value beyond conventional approaches, in support of Sustainable Development Goal (SDG) 3.4.

**Methods:**

Pre-HTN was defined according to JNC 7 guidelines. Using the recent nationally representative sample from BDHS 2022, we integrated survey-weighted bivariate analyses and multivariable logistic regression to identify independent associations. A random forest classification model was implemented to assess variable importance and predictive performance. Model evaluation was conducted using confusion matrices and receiver operating characteristic curves.

**Results:**

The study found that age and BMI were the most dominant predictors of pre-HTN, with the condition significantly more prevalent among the elderly and overweight individuals. Crucially, the crude association between diabetes and pre-HTN disappeared after multivariable adjustment. A “reversal” of the social gradient was observed, as higher education significantly increased risk (AOR = 1.26). Adults aged ≥60 years had nearly threefold higher odds compared with those aged < 30 years (AOR = 2.75; 95% CI: 2.32–3.26). Regionally, residents of the coastal region exhibited a significantly higher prevalence of elevated blood pressure (AOR: 1.19; 95% CI: 1.04–1.36) compared to the central region. The random forest model ranked age, body mass index, and sex as the most influential predictors. Discriminatory performance was modest and similar between models (AUC: logistic regression = 0.63; random forest = 0.62).

**Conclusion:**

Pre-hypertension in Bangladesh is shaped largely by aging, rising body weight, and shifting lifestyle patterns among the highly educated. Both logistic regression and random forest models pointed to the same core predictors, underscoring the stability of these findings. These results highlight the need for focused prevention strategies—supporting weight control in older adults, incorporating movement into the routines of sedentary educated populations, and tailoring programs to regions where risk is consistently higher.

## Introduction

Hypertension (HTN) is globally recognized as a primary driver of premature mortality, frequently described as a “silent killer” (1). The condition accounts for approximately 9.4 million deaths annually, a mortality burden comparable to that of major infectious diseases (2). Projections indicated that by 2025, the prevalence of HTN will surge by 60%, affecting an estimated 1.56 billion individuals worldwide (3). Contrary to common misconceptions, the majority of this burden falls upon Low- and Middle-Income Countries (LMICs), which currently shoulder two-thirds of global HTN cases (1, 4). Notably, nations within the South Asian region (SAARC) exhibit prevalence rates that exceed the global average (5).

Beyond established HTN, Pre-hypertension (pre-HTN), defined by blood pressure thresholds slightly below stage 2 HTN, contribute significantly to the global disease burden (6). This condition is implicated in nearly half of all ischemic heart disease cases and approximately 60% of stroke incidents (6). Furthermore, pre-HTN is strongly associated with the incidence of renal diseases (7). Individuals presenting with pre-HTN earlier in life face a heightened long-term risk of developing Cardiovascular Diseases (CVDs) (8, 9).

Research has further corroborated the link between elevated HTN or Stage 1 HTN and the onset of Coronary Artery Diseases (CADs) (10, 11), as well as increased cardiovascular and stroke-related mortality (12, 13). Additionally, multiple studies have confirmed a significant relationship between pre-hypertensive status and Chronic Kidney Disease (CKD) (14, 15). The etiology of elevated blood pressure involves a complex interplay of risk factors. Established modifiable determinants include unhealthy dietary habits, physical inactivity, tobacco and alcohol consumption, higher Body Mass Index (BMI), and lower socioeconomic status, while non-modifiable factors include advanced age (>65 years), family history, and comorbidities such as diabetes or kidney disease (1). Current data indicates that individuals classified as overweight face more than double the risk of developing HTN compared to those with a normal weight profile (AOR = 2.15; 95% CI: 1.98–2.34) (16). Although religious observances contribute to lower rates of alcohol consumption, the prevalence of other risk factors, such as sedentary behavior and poor nutritional habits, continues to escalate (17).

Pre-HTN, affecting approximately 25 to 50% of the adult population globally, serves as a critical precursor to clinical HTN. Evidence suggests that this transition is highly preventable; intensive lifestyle modifications can reduce the relative risk of incident HTN by 20%, while single-agent pharmacological interventions may offer a more substantial reduction of 34 to 66% (18). Despite this significant potential to mitigate the onset of non-communicable diseases (NCDs), there remains a conspicuous lack of focused research on pre-HTN within the Bangladeshi context. While the burden of HTN is well-documented, the pre-hypertensive stage, where intervention is often most effective, has not been rigorously analyzed using recent nationally representative data. Consequently, this study addresses this critical gap by investigating pre-HTN among the adult population of Bangladesh using the most recent national survey data.

Our research aims to provide a robust evidence base for early intervention by addressing two primary objectives. First, we seek to determine if the risk factors for pre-HTN are identical to those of established HTN or if they represent a distinct epidemiological profile. Second, we enhance the traditional analytical framework by integrating ensemble machine learning with standard statistical methods. This study is fundamentally aligned with United Nations Sustainable Development Goal (SDG) 3, specifically Target 3.4, which mandates a one-third reduction in pre-mature mortality from NCDs through prevention and treatment. By focusing on pre-HTN as a primary outcome—a proactive rather than reactive approach—this paper offers a strategic roadmap for early cardiovascular risk management in Bangladesh.

## Materials and methods

### Study design and setting

The research utilized data obtained from the 2022 Bangladesh Demographic and Health Survey (BDHS), which is a nationally representative cross-sectional survey serving as secondary data source. The 2022 BDHS was the ninth iteration of the survey, conducted under the authority of the National Institute of Population Research and Training (NIPORT), Medical Education and Family Welfare Division of the Ministry of Health and Family Welfare (MOHFW) in Bangladesh. The survey was implemented by Mitra and Associates, with technical assistance provided by ICF through The DHS Program and financial support provided by the United States Agency for International Development (USAID) and the Government of Bangladesh (19).

**Figure 1:**
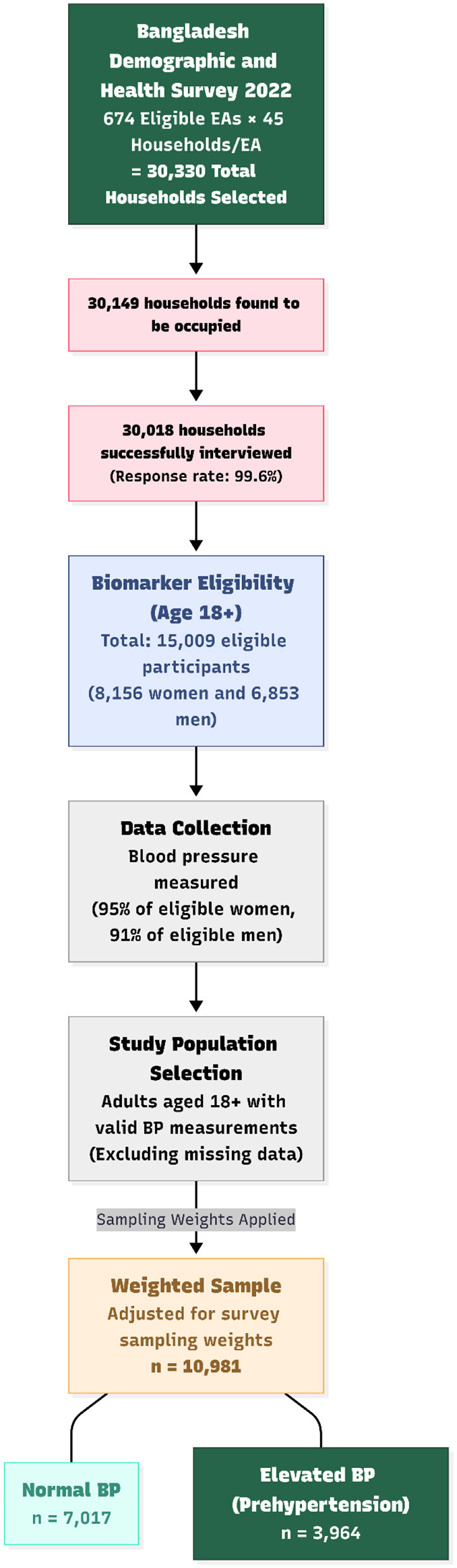
Flowchart of selecting analytic sample.

This study specifically utilized data from the Biomarker Questionnaire, one of the four instruments used in the 2022 BDHS. Anthropometric measurements and biomarker testing (blood pressure and blood glucose) were conducted in a subsample of selected households. In these households, blood pressure measurements were collected from all eligible men and women aged 18 years and older. The sample selection process is shown in Figure 1.

### Outcome measure

The primary outcome for this study was pre-HTN with binary levels (No: normotensive, Yes: elevated BP/ stage 1 HTN). Blood pressure measurements were obtained using the Multi-User Upper Arm Blood Pressure Monitor (Model UA-767F/FAC). To accommodate respondents with varying arm circumferences, field teams utilized monitors with three different cuff sizes: the UA-767F/FAC (medium cuff), UA-767PVS (small cuff), and UA-789AC (extra-large cuff). In accordance with the BDHS biomarker protocol, three blood pressure readings were recorded for each eligible respondent at intervals of approximately 5 min. The arithmetic averages of the second and third measurements were calculated to determine the final blood pressure values.

Pre-HTN was defined based on the Seventh Report of the Joint National Committee (JNC 7) guidelines (20). Respondents were classified as having pre-HTN if their systolic blood pressure ranged from 120 to 139 mmHg or their diastolic blood pressure ranged from 80 to 89 mmHg. Respondents were considered normotensive if systolic blood pressure was < 120 mmHg and diastolic blood pressure was < 80 mmHg.

### Independent variables

The selection of explanatory variables was informed by a comprehensive evaluation of relevant literature. These variables were categorized into individual, household, and community levels, covering demographic, biomedical, and behavioral characteristics.

Individual-Level Factors Individual attributes included age (categorized as < 30, 30–39, 40–49, 50–59, 60–69, and ≥70 years), sex (male, female), educational attainment (No education, Primary, Secondary, and Higher-secondary and above). Body Mass Index (BMI) was classified according to World Health Organization (WHO) standards: underweight (< 18.5 kg/m^2^), normal (18.5–24.99 kg/m^2^), overweight (25–29.99 kg/m^2^), and Obese (≥30 kg/m^2^). Diabetes status was defined following WHO classification; respondents with a fasting blood glucose ≥7.0 mmol/L (126 mg/dl) or those currently taking medication for diabetes were considered to have diabetes (21).

Household and Community-Level Factors Wealth status was assessed at the household level using Principal Component Analysis (PCA) based on ownership of assets (e.g., televisions, bicycles) and housing characteristics (e.g., drinking water sources, sanitation facilities, building materials) (22). The resulting wealth index was classified into three groups: poor, middle, and rich (23). Community-level factors included place of residence (City, Semi-urban, and Rural) and regional location. The country's administrative divisions were grouped into four regions: Coastal (Barisal, Chattogram, and Khulna), Central (Dhaka and Mymensingh), North (Rajshahi and Rangpur), and East (Sylhet).

### Statistical analysis

Data cleaning, management, and statistical analyses were conducted using Stata (Version 17.0) and R (Version 4.5.1). To ensure the findings were nationally representative, all analyses incorporated the appropriate survey weights provided by the BDHS, which adjusted for the two-stage stratified cluster sampling design and non-response rates.

Initially, descriptive statistics were generated to characterize the study population. Since all explanatory variables were categorical and the outcome was binary (Pre-HTN: yes/no), the Chi-square test of independence was performed to examine the bivariate association between each independent variable and pre-HTN. Variables found to be significant in the bivariate analysis or deemed clinically relevant based on literature were included in the multivariable model.

A survey-weighted binary logistic regression model was fitted to identify the independent risk factors associated with pre-HTN. Results were reported as Adjusted Odds Ratios (AOR) with 95% Confidence Intervals (CI). A *p*-value of < 0.05 was considered statistically significant. To address potential limitations of the parametric logistic regression model, specifically regarding potential class imbalance and complex non-linear interactions among predictors—a Random Forest classification model was employed. This machine learning approach was utilized to determine if a non-parametric, flexible model could yield superior predictive performance or reveal distinct variable importance patterns.

However, comparative analysis of model performance metrics indicated that the Random Forest model did not provide substantial improvements in predictive accuracy or discriminative ability compared to the logistic regression model. Consequently, given the Random Forest's lack of additional insight and the superior interpretability of linear models in epidemiological contexts, the logistic regression model was retained as the primary analytical method for this study.

### Logistic regression (LR)

Logistic Regression (LR) is a standard probabilistic framework utilized for binary classification tasks, estimating the likelihood of a specific outcome based on a set of input variables (24). Valued for its parsimony and high interpretability, LR is particularly effective for isolating the impact of individual predictors on health outcomes, such as pre-HTN. This model facilitates a precise understanding of the statistical significance and directional influence of each determinant.

### Random forest

Random Forest (RF) is an ensemble learning algorithm that generates a multitude of decision trees and synthesizes their individual outputs to bolster predictive precision (25). By aggregating the predictions across these various trees, RF effectively reduces the risk of overfitting and enhances the model's ability to generalize to new data. This approach is especially advantageous for managing high-dimensional datasets and capturing intricate, non-linear interactions among variables, making it a robust tool for analyzing the complex determinants of pre-HTN.

### Model evaluation and performance metrics

To assess and compare the predictive capability of the Logistic Regression and Random Forest models, a comprehensive set of evaluation metrics was employed. The performance of each model was evaluated using Accuracy, Sensitivity (Recall), Specificity, Precision, Negative Predictive Value (NPV), F1-score, and the Area Under the Receiver Operating Characteristic Curve (AUC) (26).

In addition to numerical metrics, visual tools were utilized to analyze model behavior further.

Confusion Matrices were generated for both models to visualize the distribution of True Positives, True Negatives, False Positives, and False Negatives, providing insight into the classification errors.

The Receiver Operating Characteristic (ROC) curves were plotted to illustrate the trade-off between sensitivity and specificity across different threshold settings (27). The AUC value was calculated from these curves to provide a single aggregate measure of performance.

To identify the most influential determinants of pre-HTN, the variable importance scores were extracted from the Random Forest model. These scores were visualized in a feature importance plot to rank predictors based on their contribution to the model's predictive power.

## Results

The analytic sample included adult participants with complete information on blood pressure measurements and relevant socio-demographic and health-related variables. Survey sampling weights were applied in all analyses to address the complex survey design and to ensure national representativeness. Overall, the study population comprised adults spanning a wide range of age groups, body mass index categories, educational levels, and socioeconomic strata, residing in both urban and rural areas and across all geographic regions of Bangladesh. The distribution of key characteristics according to Pre-HTN status is displayed in Table 1.

**Table 1:** Distribution of pre-hypertensive people by socio-demographic variables.

Characteristic	No (*N* = 7,017^a^)	Yes (N = 3,964^a^)	*p*-value^b^
BMI			
Underweight	635 (9.0%)	196 (5.0%)	< 0.001
Normal	2,207 (31%)	1,009 (25%)	
Overweight	812 (12%)	623 (16%)	
Obese	3,362 (48%)	2,136 (54%)	
Diabetes status			
No	6,135 (88%)	3,378 (85%)	0.001
Yes	849 (12%)	564 (15%)	
Age			
< 30	2,867 (41%)	1,061 (27%)	< 0.001
30–39	1,665 (24%)	1,008 (25%)	
40–49	1,105 (16%)	755 (19%)	
50–59	679 (9.0%)	522 (13%)	
60+	701 (10.0%)	618 (16%)	
Sex			
Female	3,829 (55%)	2,016 (51%)	< 0.001
Male	3,187 (45%)	1,948 (49%)	
Residential region			
Central	2,236 (32%)	1,076 (27%)	< 0.001
Coastal	2,305 (33%)	1,311 (33%)	
North	1,662 (24%)	1,082 (27%)	
East	813 (12%)	495 (12%)	
Wealth index			
Poor	2,878 (41%)	1,548 (39%)	0.035
Middle	1,450 (21%)	781 (20%)	
Rich	2,689 (38%)	1,635 (41%)	
Residence			
Urban	1,848 (26%)	1,036 (26%)	0.868
Rural	5,169 (74%)	2,928 (74%)	
Education			
No education, pre-school/early childhood education	1,513 (22%)	1,020 (26%)	< 0.001
Primary	1,827 (26%)	1,010 (25%)	
Secondary	2,595 (37%)	1,304 (33%)	
Higher	1,082 (15%)	631 (16%)	

a n (%);

b Pearson's χ^2^: Rao & Scott adjustment; Design-based Kruskal–Wallis test.

### Distribution of Pre-hypertension

Table 1 presents the bivariate associations between pre-HTN status and related socio-demographic and health-related characteristics. The results from chi-square test showed significant differences in the distribution of Pre-HTN across most explanatory variables. Pre-HTN prevalence differed significantly across BMI categories (*p* < 0.001), with higher prevalence among overweight and obese respondents and the lowest prevalence among underweight participants. Pre-HTN was also more prevalent among participants with diabetes compared to non-diabetic individuals (*p* = 0.001). A clear age gradient was observed, with Pre-HTN prevalence increasing gradually with age (*p* < 0.001) and reaching the highest levels among adults aged 60 years and older. Pre-HTN prevalence was significantly higher among males than females (*p* < 0.001). Regional variation was evident (*p* < 0.001), with higher prevalence observed in the coastal regions (33%) of Bangladesh.

Pre-HTN prevalence also differed across wealth categories (*p* = 0.035), with higher prevalence among individuals from wealthier households. In addition, lower levels of education were associated with a higher prevalence of Pre-HTN compared with secondary or higher education levels (*p* < 0.001). In contrast, place of residence (urban vs. rural) was not significantly associated with Pre-HTN (*p* = 0.868). Overall, bivariate analyses identified significant associations between Pre-HTN and BMI, diabetes status, age, sex, region, wealth index, and educational background. Variables demonstrating significant bivariate associations were subsequently included in the multivariable logistic regression model.

### Logistic regression

Table 2 summarizes the results of the survey-weighted multivariable logistic regression analysis examining factors independently associated with pre-HTN. After adjustment for underlying confounders, body mass index (BMI), age, sex, region of residence, and educational status remained significantly associated with the response. Compared with respondents with normal BMI, underweight individuals had significantly lower odds of pre-HTN (adjusted odds ratio [AOR] = 0.66; 95% CI: 0.55–0.78), whereas overweight (AOR = 1.61; 95% CI: 1.40–1.86) and obese individuals (AOR = 1.97; 95% CI: 1.54–2.52) had significantly higher odds. Diabetes status was not significantly associated with outcome after adjustment (*p* = 0.559). Age showed a strong positive association with Pre-HTN. Relative to individuals younger than 30 years, the odds increased gradually across age groups: 30–39 years (AOR = 1.66; 95% CI: 1.46–1.88), 40–49 years (AOR = 1.94; 95% CI: 1.69–2.22), 50–59 years (AOR = 2.38; 95% CI: 2.01–2.82), and 60 years and older (AOR = 2.75; 95% CI: 2.32–3.26).

**Table 2:** Survey-weighted multivariable logistic regression analysis of factors associated with pre-hypertension in Bangladesh (BDHS 2022).

Characteristic	AOR (95% CI)	*p*-value
Body mass index (BMI)		
Normal	Ref	< 0.001
Underweight	0.66 (0.55, 0.78)	
Overweight	1.61 (1.40, 1.86)	
Obese	1.97 (1.54, 2.52)	
Diabetes status		
No	Ref	0.559
Yes	1.04 (0.91, 1.18)	
Age (years)		
< 30	Ref	< 0.001
30–39	1.66 (1.46, 1.88)	
40–49	1.94 (1.69, 2.22)	
50–59	2.38 (2.01, 2.82)	
60+	2.75 (2.32, 3.26)	
Sex		
Female	Ref	< 0.001
Male	0.58 (0.46, 0.74)	
Residential region		
Central	Ref	0.004
Coastal	1.19 (1.04, 1.36)	
North	1.28 (1.11, 1.48)	
East	1.22 (1.04, 1.42)	
Wealth index		
Poor	Ref	0.198
Middle	1.01 (0.89, 1.14)	
Rich	1.10 (0.99, 1.23)	
Educational attainment		
No education/pre-school	Ref	0.007
Primary	**0.97** (0.85, 1.11)	
Secondary	1.03 (0.90, 1.17)	
Higher	1.26 (1.07, 1.49)	

CI, confidence interval; OR, odds ratio.

Sex was also significantly associated with Pre-HTN, with males having lower odds compared with females (AOR = 0.58; 95% CI: 0.46–0.74). Regional variations were observed, as individuals living in coastal (AOR = 1.19; 95% CI: 1.04–1.36), northern (AOR = 1.28; 95% CI: 1.11–1.48), and eastern regions (AOR = 1.22; 95% CI: 1.04–1.42) had higher odds of Pre-HTN compared with those residing in the central region of the country. Although household wealth index was not significantly associated with Pre-HTN after adjustment (*p* = 0.198), educational background remained significant. Compared with individuals with no education or pre-school-level education, individuals with higher education had increased levels of Pre-HTN (AOR = 1.26; 95% CI: 1.07–1.49), while primary and secondary education levels were not significantly associated.

### Random forest

**Figure 2:**
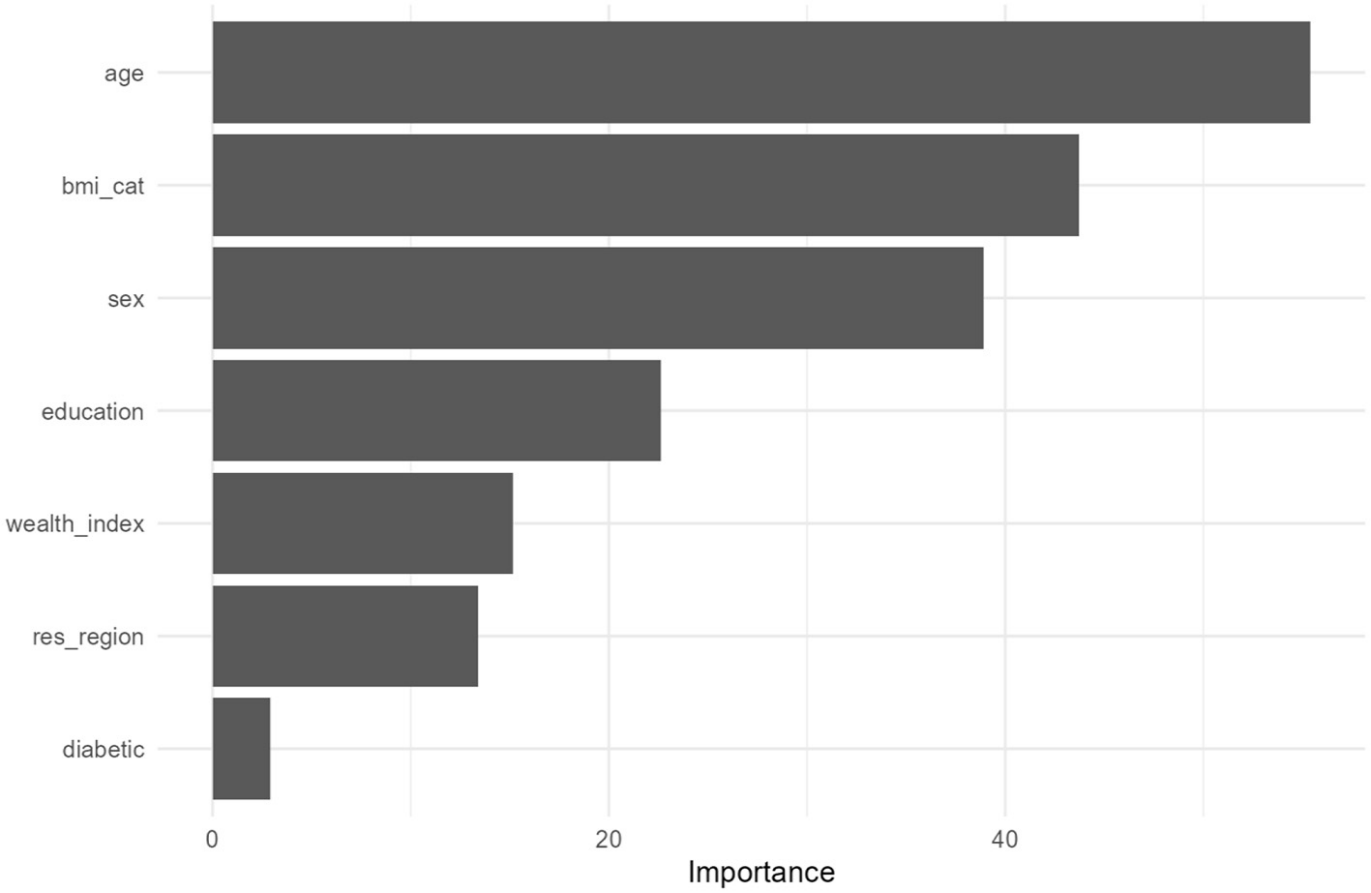
Variable importance plot.

Figure 2 presents the variable importance plot obtained from the random forest model, explaining the relative contribution of each predictor to the classification of outcome. Age was identified as the most influential predictor, showing the highest importance score among all predictor variables. This was followed by BMI category and sex, indicating that demographic and anthropometric characteristics were the primary contributors to model predictions. Educational status demonstrated a moderate level of importance, whereas wealth index and region of residence contributed comparatively less to the model's predictive performance. Diabetes status showed the lowest importance among the included predictors. Overall, the variable importance pattern observed in the random forest model was similarly consistent with the findings from the multivariable logistic regression analysis, particularly with respect to the important role of age, BMI, and sex. To assess how these predictors were translated into classification performance, confusion matrices for both models were examined.

### Predictive performance

**Figure 3:**
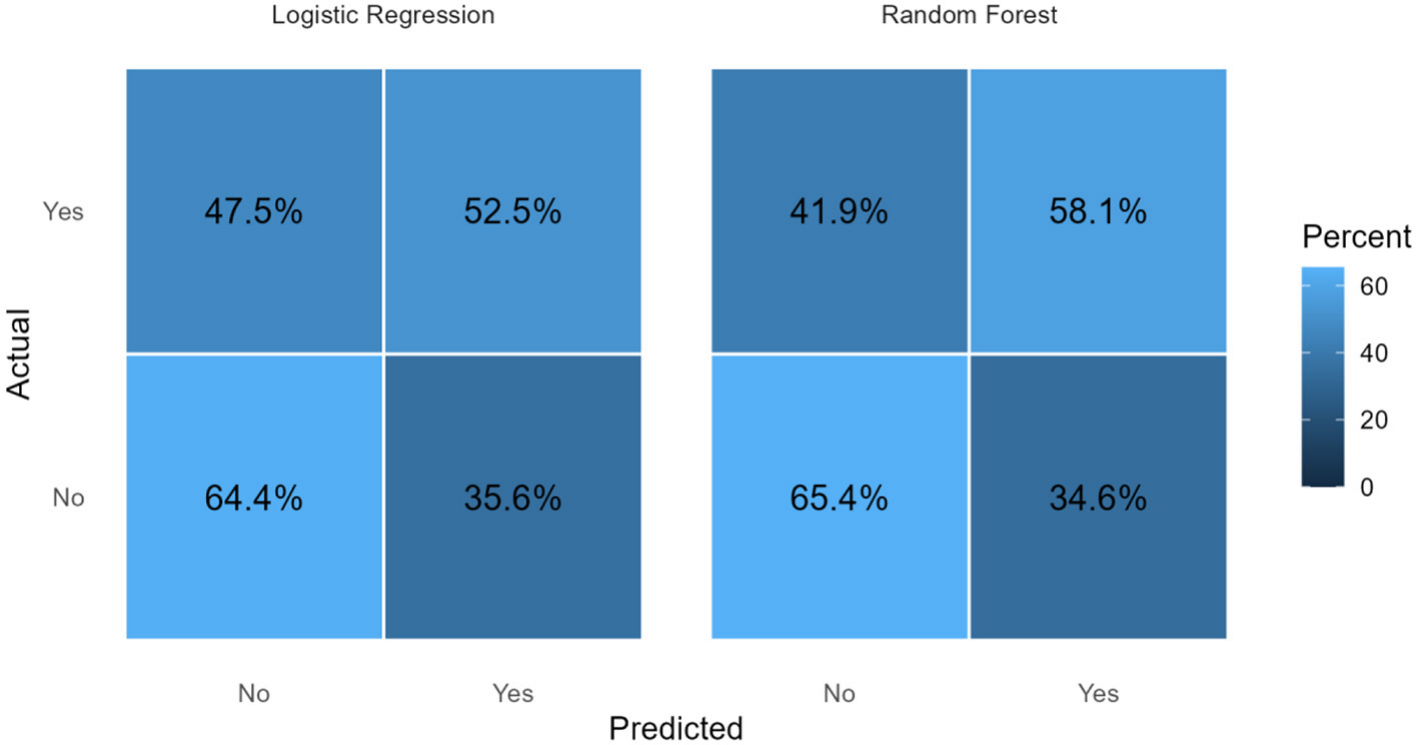
Confusion matrices.

Figure 3 presents the confusion matrices (row percentages) for LR and RF models. For both approaches, correct classification of respondents without Pre-HTN (“No”) was higher than correct identification of those with outcome (“Yes”). In the logistic regression model, 64.4% of non–pre-hypertensive individuals were correctly classified, while 52.5% of pre-hypertensive individuals were correctly identified. Similarly, the random forest model correctly classified 65.4% of non–pre-hypertensive individuals and 58.1% of pre-hypertensive individuals. Overall, the RF model showed modestly improved classification of Pre-HTN compared with logistic regression, while performance in identifying respondents without the condition was comparable between the two models.

**Figure 4:**
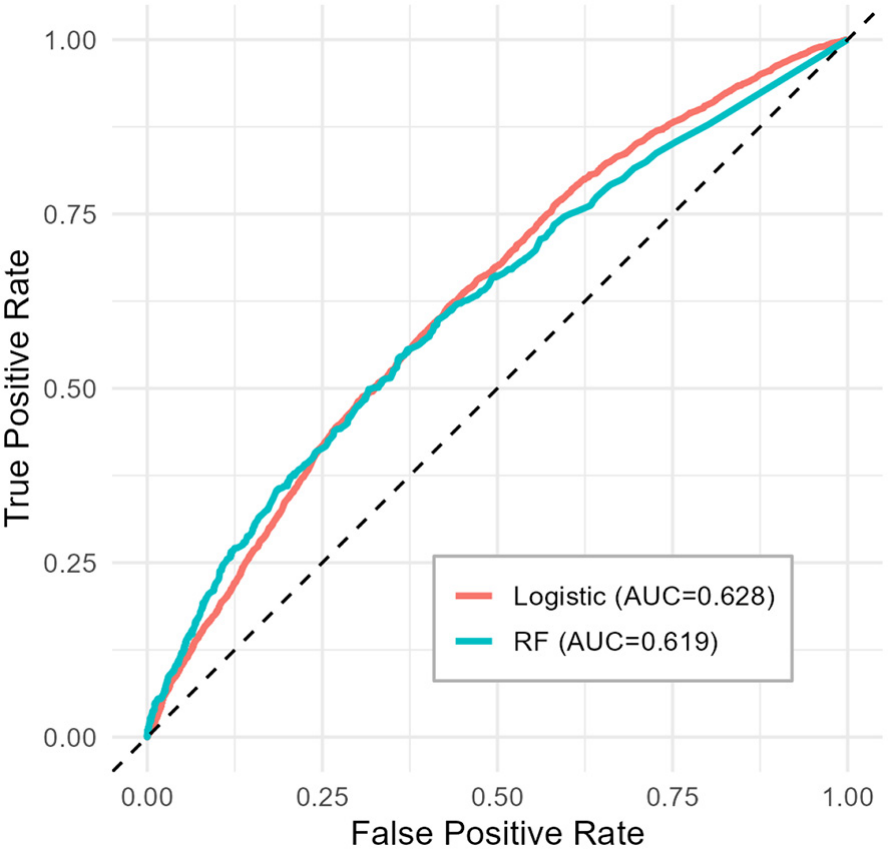
Receiver operating characteristic (ROC) curves.

Figure 4 compares the receiver operating characteristic (ROC) curves of the LR and RF models. Both models showed modest discriminatory ability for identifying Pre-HTN, with area under the curve (AUC) values of 0.628 for logistic regression and 0.619 for the random forest model. The ROC curves largely overlapped across the range of false positive rates, which indicates similar overall discrimination. Although logistic regression showed a slightly higher AUC, the difference in discriminatory performance between the two approaches was minimal. To further compare model performance across multiple evaluation criteria, additional classification metrics were examined.

**Figure 5:**
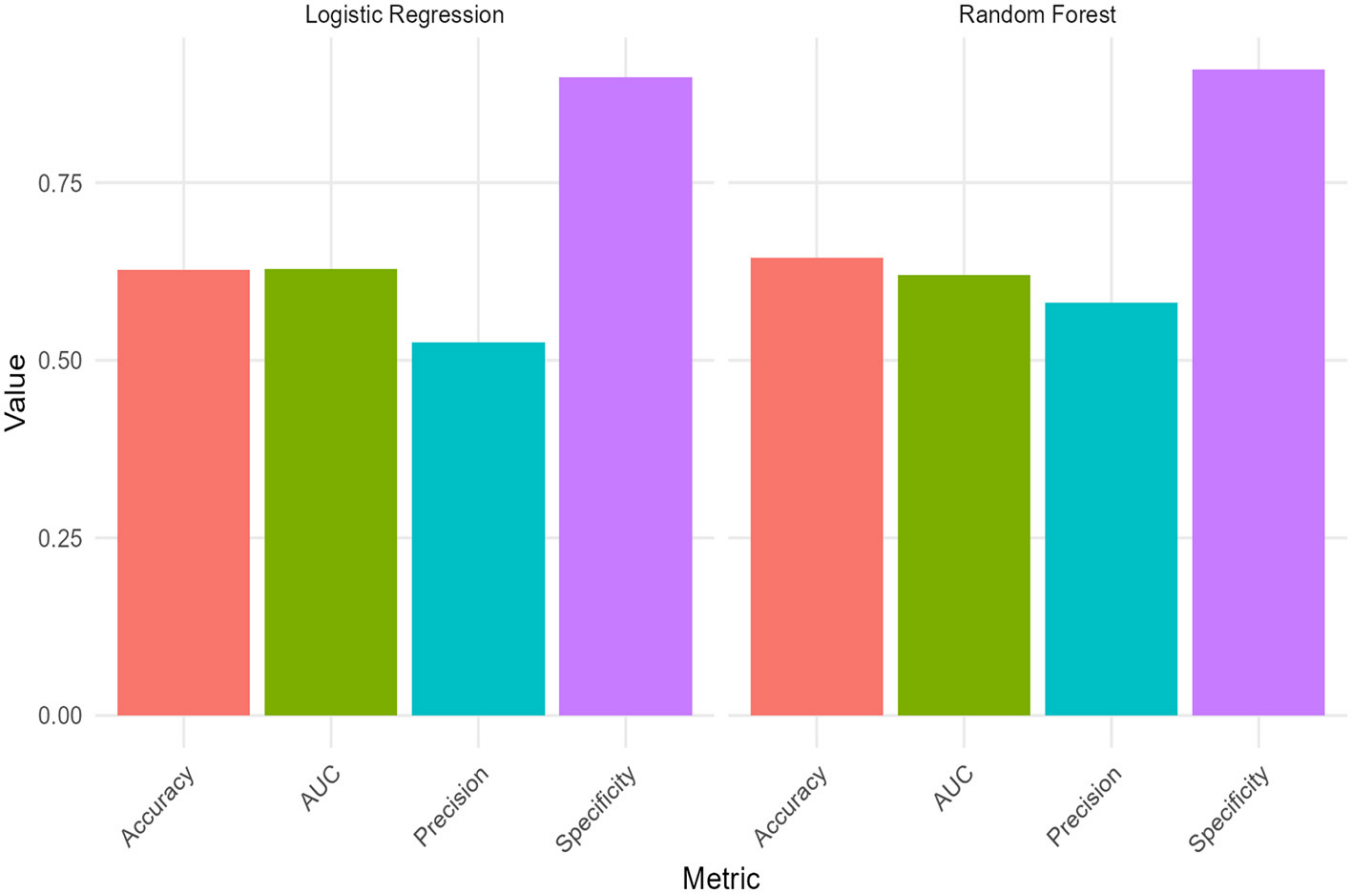
Predictive performances of LR and RF.

Figure 5 summarizes the evaluation metrics for the logistic regression and random forest models. Overall accuracy was comparable between the two approaches, with both models achieving moderate accuracy in classifying Pre-HTN status. The logistic regression model showed slightly higher precision, whereas the random forest model demonstrated marginally better specificity. AUC values were similar for both models, indicating comparable discriminatory performance.

Taken together, these findings indicate that although the random forest model provided modest improvements in certain classification metrics, overall performance was broadly similar between the two modeling approaches.

## Discussion

This study provides a nationally representative assessment of pre-hypertension among adults in Bangladesh using BDHS 2022. Combining survey–weighted logistic regression with an ensemble machine learning approach, we identified key socio-demographic and anthropometric factors associated with pre-HTN. RF was used as a non-parametric robustness check to cross-validate the regression findings and rank variable importance; its results converged with logistic regression, indicating similar insight and supporting the stability of the identified predictors in this population. Although, RF added a complementary perspective on variable importance but did not improve discrimination over logistic regression (AUC ~0.62–0.63, overlapping ROC curves). In structured survey data with a limited, well-defined predictor set, this equivalence supports using the interpretable regression model as primary.

Age showed a clear, monotonic association with pre-hypertension and was the top-ranked variable in RF. Adjusted odds rose across age groups, reaching the highest levels among older adults, consistent with established age-related vascular changes and with prior BDHS analyses documenting higher hypertension burden at older ages (28, 29). BMI was likewise a strong predictor; overweight and obesity were associated with higher odds, whereas underweight was associated with lower odds relative to normal BMI. These findings align with regional literature citing adiposity-linked hemodynamic and metabolic pathways in elevated blood pressure (5, 30). Sex differences persisted after adjustment, with males exhibiting lower odds than females. This reversal relative to the crude distribution underscores confounding by age and adiposity and illustrates why adjusted comparisons are essential (31). Mechanisms cannot be resolved here because relevant behaviors were not directly measured.

For diabetes, the bivariate association did not persist after adjustment, and diabetes ranked lowest in RF importance. These convergent signals suggest that the crude correlation was largely shared with age and BMI rather than an independent relationship in the pre-hypertensive range. Cohort evidence has reported stronger associations at higher systolic thresholds (e.g., ≥130 mmHg), while 120–129.9 mmHg elevations were not independently predictive (32). We therefore interpret our cross-sectional results as compatible with confounding by shared cardiometabolic profiles rather than as evidence of a direct effect at pre-hypertensive levels.

Educational attainment remained associated with higher odds of pre-hypertension for the highest education group, whereas the household wealth index was not significant after adjustment. In the context of South Asian epidemiologic transition, similar “reversal” of the social gradient has been described, but because BDHS 2022 lacks direct measures of physical activity, diet, and work patterns, this analysis cannot identify the operative pathways (29, 33–35). Regional differences persisted after adjustment, with higher odds in coastal, northern, and eastern regions relative to the central region. These findings are consistent with prior reports from Bangladesh; however, determinants specific to place (e.g., environment, diet, services) were not measured here, so causal interpretation is not attempted (36, 37).

From a public-health standpoint, these results support early, population-level strategies that emphasize weight management, diet quality, physical activity promotion, and routine BP screening—especially as adults enter midlife and in regions with consistently higher odds. While these are standard recommendations for preventing progression to clinical hypertension, their prioritization here follows directly from the observed age- and BMI-linked patterns and regional heterogeneity.

Limitations include the cross-sectional design (limiting causal inference and precluding assessment of progression), single-visit BP measurement, and incomplete measurement of key behaviors and structural exposures (diet, salt intake, quantifiable physical activity, tobacco, and psychosocial stress), leaving room for residual confounding—even with survey design properly accounted for.

## Conclusion

This study fills an important evidence gap by providing a nationally representative profile of pre-HTN risk in Bangladesh using the BDHS 2022 dataset. The results highlight advancing age and higher BMI as the strongest predictors of pre-hypertension. Additionally, the adjusted analysis revealed a noteworthy sex difference, with males showing lower odds of pre-HTN compared to females. The study also documents a “reversal” of the expected social gradient: individuals with higher educational attainment faced increased risk, likely reflecting a shift toward sedentary, white-collar occupations. Evident regional disparities were also observed, with adults in the coastal, northern, and eastern regions experiencing significantly higher odds of blood pressure. Importantly, once major socio-demographic factors were accounted for, the crude association between diabetes and pre-HTN was no longer evident, suggesting that shared metabolic pathways, particularly aging and obesity, drive this relationship rather than a direct effect of early blood pressure elevation on glycemic control.

Methodologically, the use of an ensemble machine learning approach complemented the traditional survey-weighted logistic regression by overcoming potential parametric constraints and providing a robust assessment of variable importance. The alignment of both methods, demonstrated through comparable predictive performance and identification of the same core determinants, underscores the stability and reliability of these findings across analytical frameworks.

These insights highlight the need for national health strategies to move beyond broad awareness campaigns toward targeted, preventative interventions. Key priorities include promoting weight management and routine blood pressure screening among adults entering midlife. Public health initiatives should also adapt to the lifestyle patterns of the educated workforce by integrating physical activity into sedentary daily routines, while tailoring region-specific strategies—such as enhanced sodium-reduction programs in coastal areas. Through such targeted measures, pre-hypertension can be reframed as a crucial opportunity for early cardiovascular disease prevention in Bangladesh.

## Data Availability

Publicly available datasets were analyzed in this study. This data can be found here: https://dhsprogram.com/data/available-datasets.cfm.
